# Panic or peace – prioritising infant welfare when medicating feverish infants: a grounded theory study of adherence in a paediatric clinical trial

**DOI:** 10.1186/s12887-022-03230-4

**Published:** 2022-04-11

**Authors:** Eunicia Tan, Karen Hoare, Judith Riley, Kathryn Fernando, Libby Haskell, Christopher JD McKinlay, Stuart R Dalziel, Irene Braithwaite

**Affiliations:** 1 Department of Surgery, Faculty of Medical and Health Sciences, University of Auckland, Auckland, New Zealand; 2 Emergency Department, Middlemore Hospital, Auckland, New Zealand; 3 School of Nursing, Massey University, Auckland, New Zealand; 4 Greenstone Family Clinic, Manurewa, Auckland, New Zealand; 5 Medical Research Institute of New Zealand, Wellington, New Zealand; 6 Children’s Emergency Department, Starship Children’s Hospital, Auckland, New Zealand; 7 Liggins Institute, University of Auckland, Auckland, New Zealand; 8 Kidz First Neonatal Care, Counties Manukau Health, Auckland, New Zealand; 9 Department of Paediatrics: Child and Youth Health, Faculty of Medical and Health Sciences, University of Auckland, Auckland, New Zealand

**Keywords:** Paracetamol, Ibuprofen, Paediatric, Constructivist grounded theory, Trial adherence, Fever phobia, Trust, COVID-19

## Abstract

**Background:**

Literature on factors influencing medication adherence within paediatric clinical trials is sparse. The Paracetamol and Ibuprofen in the Primary Prevention of Asthma in Tamariki (PIPPA Tamariki) trial is an open-label, randomised controlled trial aiming to determine whether paracetamol treatment, compared with ibuprofen treatment, as required for fever and pain in the first year of life, increases the risk of asthma at age six years. To inform strategies for reducing trial medication crossovers, understanding factors influencing the observed ibuprofen-to-paracetamol crossovers (non-protocol adherence) is vital. The aim of this study was to investigate the factors influencing the decision-making process when administering or prescribing ibuprofen to infants that may contribute to the crossover events in the PIPPA Tamariki trial.

**Methods:**

Constructivist grounded theory methods were employed. We conducted semi-structured interviews of caregivers of enrolled PIPPA Tamariki infants and healthcare professionals in various healthcare settings. Increasing theoretical sensitivity of the interviewers led to theoretical sampling of participants who could expand on the teams’ early constructed codes. Transcribed interviews were coded and analysed using the constant comparative method of concurrent data collection and analysis.

**Results:**

Between September and December 2020, 20 participants (12 caregivers; 8 healthcare professionals) were interviewed. We constructed a grounded theory of *prioritising infant welfare* that represents a basic social process when caregivers and healthcare professionals medicate feverish infants. This process comprises three categories: *historical*, *trusting relationships* and *being discerning*; and is modified by one condition: *being conflicted*. Participants bring with them historical ideas. Trusting relationships with researchers, treating clinicians and family play a central role in enabling participants to challenge historical ideas and be discerning. Trial medication crossovers occur when participants become conflicted, and they revert to historical practices that feel familiar and safer.

**Conclusions:**

We identified factors and a basic social process influencing ibuprofen use in infants and trial medication crossover events, which can inform strategies for promoting adherence in the PIPPA Tamariki trial. Future studies should explore the role of trusting relationships between researchers and treating clinicians when conducting research.

**Supplementary Information:**

The online version contains supplementary material available at 10.1186/s12887-022-03230-4.

## Background

Paracetamol and ibuprofen are the most widely prescribed and available over-the-counter medications for the management of fever and pain in children. Although previous systematic reviews have shown paracetamol and ibuprofen to be equivalent in terms of antipyretic and analgesic efficacy and safety [1–5], paracetamol is commonly considered first-line because its safety is perceived to be more assured [3, 6, 7]. Recently, there has been a growing body of research suggesting an association between paracetamol use in infancy and the development of wheezing and asthma [8–10]. We are conducting the Paracetamol and Ibuprofen in Primary Prevention of Asthma in Tamariki (PIPPA Tamariki) trial to determine whether use of paracetamol for fever and pain in the first year of life, compared to ibuprofen, increases the risk of asthma and atopic disease in childhood [11].

PIPPA Tamariki is a multicentre, open-label, two-arm parallel group randomised controlled trial (RCT) being conducted in New Zealand, with participants randomised to paracetamol or ibuprofen as required for fever or pain [11]. Our sample size of 3,922 allows for an efficacy dilution factor of 10% due to participants being exposed to the alternative intervention (henceforth referred to as trial medication “crossover”) within the first year of life. Since trial commencement in April 2018, we have observed a threefold higher rate of ibuprofen-to-paracetamol crossovers (participants randomised to ibuprofen exposed to paracetamol; 36%) than paracetamol-to-ibuprofen crossovers (participants randomised to paracetamol exposed to ibuprofen; 12%). The higher-than-anticipated crossover rate threatens the internal validity of the and has implications for the trial’s statistical power, increasing the risk of a Type II error [12, 13].

The current coronavirus (COVID-19) pandemic may have additionally influenced the use of paracetamol and ibuprofen among participants. On 18 March 2020, the World Health Organization (WHO) issued a statement recommending people with COVID-19 symptoms avoid taking ibuprofen [14]; however, this was subsequently retracted following a systematic review of the evidence [15, 16], which did not identify any adverse effects. Despite these retractions, the initial recommendations cautioning against ibuprofen use may have contributed to a perception that paracetamol should be used in preference to ibuprofen, although the effects of these statements on caregivers of participants remain unclear.

Studies addressing participation in RCTs have mainly focused on strategies to improve recruitment [17–19] and retention [19, 20]. Few studies have examined enhancing medication adherence in research [12]. One adherence enhancement strategy that has been suggested is to seek an understanding of participants’ motivations for non-adherence [12]. Although there is literature describing the use of paracetamol and ibuprofen among caregivers [21, 22], no previous studies have explored the underlying reasons for using one drug over the other, and none have addressed medication adherence in RCTs involving infants. Further, there is no literature on the impact of the COVID-19 pandemic on trial medication adherence. To inform strategies for reducing the crossover rate in the PIPPA Tamariki trial, we sought to gain an understanding of the factors surrounding medication crossover events by exploring these events through a qualitative lens.

The primary aim of this study was to investigate the factors influencing the decision-making process when administering or prescribing ibuprofen to infants that may contribute to the crossover events in the PIPPA Tamariki trial. Secondary aims were to explore the impact of the 18 March 2020 WHO statement on the attitudes, beliefs and behaviours surrounding ibuprofen use in infants.

## Methods

### Study design

This qualitative study was underpinned by Charmaz’s constructivist grounded theory methodology [23]. This approach was chosen because grounded theory seeks to generate a theoretical explanation for a basic social process influenced by a diverse set of perspectives [23, 24], thus well-suited to exploring the perspectives of both caregivers and healthcare professionals (HCPs).

The Standards for Reporting Qualitative Research (SRQR) have been followed (Additional file 1) [25]. The New Zealand Northern A Health and Disability Ethics Committee approved the study (17/NTA/233/AM04).

### Study setting and researcher characteristics

PIPPA Tamariki is a multicentre RCT being conducted at three recruitment sites in two New Zealand regions (Auckland: two sites; Wellington: one site) [11]. The research team for this grounded theory study comprised mostly of PIPPA Tamariki researchers: ET is an emergency physician involved with recruitment, follow-up and liaison with caregivers and HCPs when trial medication crossovers occur; JR and KF are research coordinators involved with recruitment and follow-up; KH is a paediatric primary care nurse practitioner (NP) and University Professor with extensive experience in grounded theory; LH is a paediatric emergency NP who has completed qualitative studies using interviews; CJDM and IB are site principal investigators; and SRD is the coordinating principal investigator of PIPPA Tamariki.

### Participants and sampling

Participants included caregivers of enrolled PIPPA Tamariki infants and HCPs in the Auckland and Wellington regions.

Caregivers were eligible for inclusion if their infant was randomised to the ibuprofen treatment arm prior to the 18 March 2020 WHO statement. HCPs were eligible for inclusion if they were actively engaged in clinical practice providing health care to infants, regardless of whether they had provided care for a PIPPA Tamariki infant previously. Temporary nursing agency staff or medical *locum* were excluded.

We used a stratified purposive sampling strategy, with a goal of recruiting participants from five participant subgroups: caregivers of PIPPA Tamariki infants who have had an ibuprofen-to-paracetamol crossover (“crossover”) before 18 March 2020; caregivers of PIPPA Tamariki infants who have NOT had an ibuprofen-to-paracetamol crossover (“non-crossover”) before 18 March 2020; community-based HCPs (midwives and well-child providers); primary care HCPs (general practitioners (GPs), practice nurses); hospital-based HCPs (emergency department (ED) and hospital doctors and nurses). This sampling strategy was designed to achieve a maximum variation sample to address the increasing theoretical sensitivity of the researchers, to ensure sufficient participants to theoretically sample following constant comparative analyses of early interviews, and to capture the broadest possible range of caregiver and HCP characteristics and experiences for data triangulation [26].

### Recruitment

A list of caregivers of PIPPA Tamariki infants who met the inclusion criteria was extracted from the trial database and stratified by recruitment site. We recruited an initial purposive sample of two crossovers and two non-crossovers per site. We recruited GPs and practice nurses from practices which were already in the PIPPA Tamariki trial database. We recruited midwives, well-child providers, and hospital-based doctors and nurses through department heads or individual approach. Caregivers of PIPPA Tamariki infants were offered a $20 fuel voucher for their time. No incentives were offered to HCPs. Participants completed a written consent form and subsequently gave verbal confirmation at the start of the interview.

### Data collection

Interviews were conducted face-to-face, by videoconference or by telephone, according to participant preference. Three researchers (ET, LH, JR) conducted the interviews. All interviews were undertaken in English, digitally recorded and transcribed verbatim after de-identification. Checked transcripts were uploaded into NVivo 12 (QSR International Pty Ltd, Chadstone, VIC, Australia) to facilitate data management and analysis.

We developed an open-ended, semi-structured interview guide (Additional file 2), allowing for exploratory questions, and informed by the research team’s theoretical sensitivity [24] surrounding potential factors that influence ibuprofen use in infants and trial medication crossover events, gained from prior conversations with caregivers and HCPs as part of PIPPA Tamariki trial procedures. The interview guide was piloted with a PIPPA Tamariki caregiver, a hospital-based nurse and a GP, with no changes made to the interview guide. These interviews were not analysed.

### Data analysis

The unit of analysis is a trial medication crossover event. Analysis followed Charmaz’s constructivist grounded theory approach [23, 24, 27], supervised by KH. Initial coding of all transcripts was performed by ET, with KH assisting with the first two transcripts to discuss ET’s increasing theoretical sensitivity and identify lines of inquiry to pursue in subsequent interviews. One other researcher (JR or KF) independently coded subsequent transcripts, so that each transcript was independently coded by two researchers. Transcripts were coded mainly using gerunds (verbs used as nouns) as they foster the examination of enacted processes suggesting that attention to actions and processes rather than individuals may aid in the process of constructing theory [23]. Constant comparative methods [28] led to codes being elevated to focused codes and to category building. Constructed codes, concepts and categories were discussed until consensus was reached. A codebook was kept and updated after each meeting to enhance trustworthiness of data analysis [29].

Data collection, analysis and theory construction proceeded concurrently, an approach considered integral to grounded theory [24]. Throughout the research process, ET wrote theoretical memos, informal analytic notes that form part of the data and are a contemporaneous record of the researcher’s developing theoretical sensitivity. Memos also serve as an audit trail and increase the trustworthiness of theoretical constructions [23, 24, 27, 30]. We planned to select additional participants based on theoretical sampling, one of the main tenets of grounded theory methodology whereby participants are purposively sampled based on constructed codes and categories and the researchers’ evolving abstractions of ‘what was going on’ in the data [31]. Our concurrent data collection and analysis identified clear early codes and categories that directed our lines of inquiry in subsequent interviews. Theoretical saturation (when no new codes or categories were constructed in three successive interviews) was reached using data from our initial purposive sample, without the need to recruit additional participants.

## Results

### Participants

Interviews were conducted between September and December 2020. Participant characteristics are shown in Table 1. Theoretical saturation was reached after 20 interviews (14 by videoconference; 4 face-to-face; 2 by telephone). Interviews lasted 27 min on average (range 14-53 min).

**Table 1:** Participant characteristics

**Region**	**Site**	**Participant subgroup**					**Total**
		Caregiver Crossover	Caregiver Non-crossover	HCP Community-based	HCP Primary care	HCP Hospital-based	
Auckland	A	2	2		Senior GP*	Senior ED nurse^a^	6
Auckland	B	2	2	Midwife		Senior ED doctor^a^	6
Wellington	C	2	2	Well-child nurse	Practice nurse Nurse practitioner	Paediatric ward nurse	8
	**Total**	**6**	**6**	**2**	**3**	**3**	**20**

*ED* Emergency department, *HCP* Healthcare professional, *GP* General practitioner

^a^Senior – healthcare professional in a senior clinical role (>10 years postgraduate experience)

### The grounded theory: *Prioritising Infant Welfare*

**Fig. 1:**
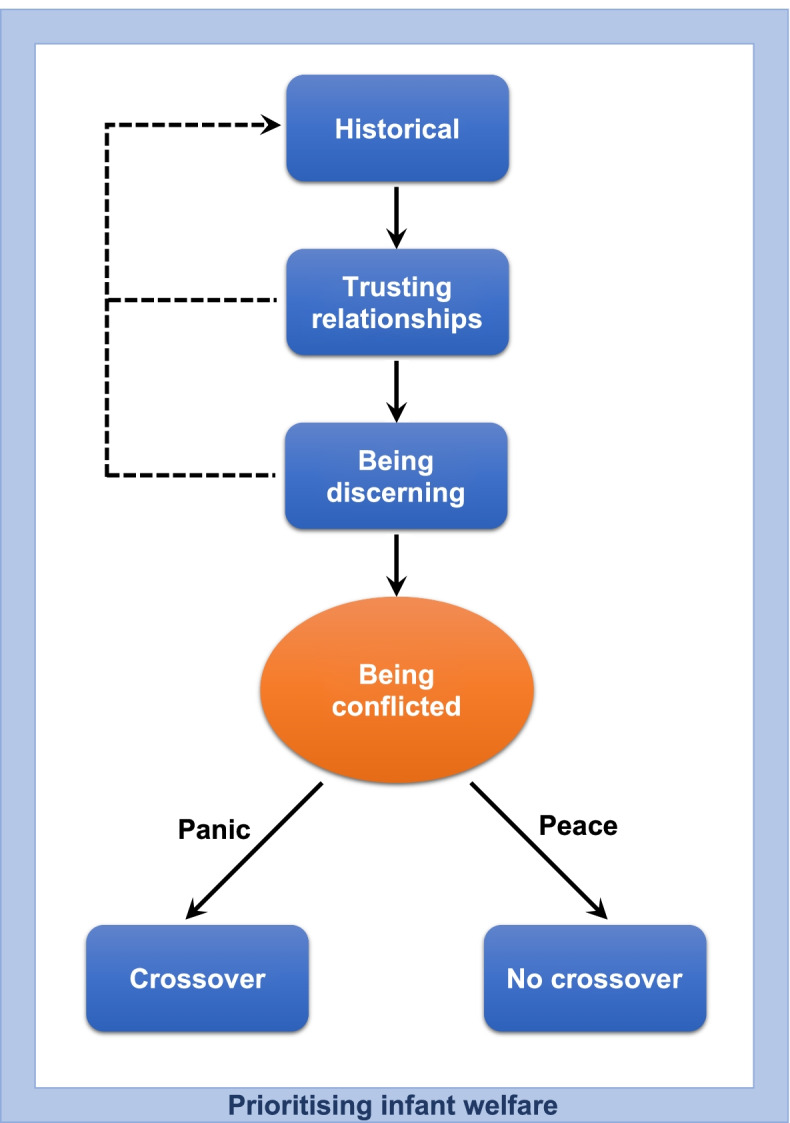
A conceptual model of the grounded theory of *prioritising infant welfare.* When medicating feverish infants, both caregivers and healthcare professionals are *prioritising infant welfare.* This basic social process is predicated upon three categories: historical, trusting relationships and being discerning; and is modified by one condition: being conflicted

When medicating feverish infants, the core category and basic social process that occurs for both caregivers and HCPs is *prioritising infant welfare,* illustrated in Figure 1.

Overwhelmingly, caregivers wanted “*to do what’s best for baby*” (Caregiver/Site B/Crossover1; Caregiver/Site C/Non-crossover1). Among HCPs, there is a strong emphasis on safe practice and following guidelines. A memo recorded ET’s observation that crossover events are also underpinned by the basic social process of prioritising infant welfare:

*When there is a conflict, participants cling to the familiar and revert to historical practices, such as alternating or combined antipyretics. They “breakdown” and “give in” because this feels like the safer course of action, based on the assumption that these practices are of benefit to the infant. By doing so, it all goes back to the premise of prioritising infant welfare.* (Memo/3 March 2021/ET)

This process is predicated upon three categories: historical, trusting relationships and being discerning; and is modified by one condition: being conflicted. Table 2 below provides an overview of the codes, categories and conditions that were abstracted to inform our theory. Additional file 3 expands on the codes and categories. We present the theory as a storyline [24] which is a strategy for facilitating integration, construction and presentation of research findings. We use data segments and memos to support the three constructed categories.

**Table 2:** Codes and categories contributing to the grounded theory of *prioritising infant welfare*

**Focused codes**									
Lack of knowledge	Fever phobia	“Paracetamol is the go-to”	Trusting the research process	Trusting healthcare professionals		Being accepting	Raising awareness	Being confident	Being discerning about COVID-19
**Categories**									
Historical			Trusting relationships				Being discerning		
***Condition: Being conflicted***									
Seeing a sick baby					Feeling pressured				
**Constructed grounded theory**									
Prioritising infant welfare									

### Historical

Caregivers and HCPs alike bring with them historical ideas and preconceptions regarding fever management and the use of medications for fever or pain in infants. Historical ideas and preconceptions were often passed down from the participants’ parents and “*older generation*” (Caregiver/Site B/Non-crossover1), or from senior colleagues during training or role-modelling in clinical practice.

*I think, yeah historically paracetamol has won*. (HCP/Community-based/Midwife)

Three focused codes comprised the historical category (Table 2; Additional file 3: Table S1).

#### Lack of knowledge

Both caregivers and HCPs showed a lack of up-to-date knowledge regarding the properties of paracetamol and ibuprofen in general, and their appropriate use in infants. Some caregivers and HCPs were unaware that ibuprofen can be used for infants [5].

*I’d heard about it* [ibuprofen]*, just not for like really small babies*. (Caregiver/Site B/Crossover1)

Some caregivers were under the impression that one drug was more effective than the other, even though the antipyretic and analgesic effects of ibuprofen and paracetamol are similar [5].

*For me Brufen seemed to work better than Pamol*. (Caregiver/Site A/Crossover2)

*I think the paracetamol’s better for fever*. (Caregiver/Site A/Crossover1)

HCPs were not immune to the lack of knowledge; some described engaging in historical practices that are no longer considered evidence-based, such as giving antipyretics routinely after childhood vaccinations [32–34].

*We have a standing order for paracetamol to be given post-immunisation*. (HCP/Primary care/Practice nurse)

There is a notion among some participants that compared to paracetamol, ibuprofen was “*more of a serious medicine*” (Caregiver/Site C/Non-crossover2), a “*proper medicine*” (HCP/Community-based/Well-child nurse), or “*stronger than the basic paracetamol that doctors give*” (Caregiver/Site B/Non-crossover2). One HCP described the lack of knowledge they observed regarding the two medications, stemming from historical use of paracetamol:

*I feel like paracetamol has been around for so long that we use it really blasé and we don’t, we don’t put enough emphasis on the damage that it probably could do. But for some reason, with ibuprofen, people kind of see that as more of a, like it could be more dangerous. I guess because it’s like a non-steroid*[al] *and that side of it...* (HCP/Community-based/Midwife)

#### Fever phobia

Fever phobic sentiments were commonplace. Both caregivers and HCPs frequently suggested that “*you have to break the fever*” (HCP/Hospital-based/Senior ED nurse) or “*bring* [the] *temperature down*” (Caregiver/Site C/Crossover2). Participants often specified a temperature cut-off that would indicate the need to administer antipyretics:

*…over three months, if the child comes in with a temperature that’s like 39 degrees* [Celsius]*, I would tend to give them some antipyretics*. (HCP/Hospital-based/Senior ED doctor)

When asked why they were concerned about a fever, participants stated that “*convulsions were* [their] *biggest worry*” (Caregiver/Site A/Crossover2), even though there is little evidence that prophylactic administration of antipyretics prevents febrile seizures in children [35, 36].

*I don’t like kids to get too hot because of febrile convulsions*. (HCP/Primary care/Senior GP)

In an effort to reduce fevers, caregivers described administering alternating or combined doses of paracetamol and ibuprofen to maintain apyrexia, a practice often learned from their HCPs, and at times leading to incorrect dosing.

*And so, after going to the doctors… I think from that point* [the doctor] *wanted to give usPamolbut we said we were in the ibuprofen* [group] *so they gave us both. So, we started giving him like 3 ml of one and 2 ml of the other instead of just 5 ml of one of them. *(Caregiver/Site B/Non-crossover1)

#### “Paracetamol is the go-to”

This *in vivo* code represents the widely held belief that paracetamol is the first-line for treating fever or pain in infants, because “*paracetamol generally cuts the mustard*” (HCP/Primary care/NP), and “*it seems to be like the answer to everything*” (Caregiver/Site C/Non-crossover2).

*Paracetamol’s my go-to and it tends to work well for infants I’ve used it for*. (HCP/Primary care/NP)

*…if Iwas to talk to one of my friends and said, oh you know, and said* [child] *got a fever – they’d say, give herPamol*. *It’s just the go-to thing*. (Caregiver/Site A/Non-crossover2)

*I would usually always recommend paracetamol as a first-line and then if that didn’t work, then I would look into using ibuprofen after the fact.* (HCP/Primary care/Practice nurse)

One potential reason for paracetamol being considered first-line is brand recognition. “*Pamol*”, one of the brand names for paracetamol in New Zealand, has become a household name.

*…I think it’s because Pamol was such a prominent brand for babies, whereas, ibuprofen wasn’t.* (Caregiver/Site A/Crossover1)

*And you know, because Pamol’s been prescribed for so many years, I think people just gravitate to Pamol as the medication of choice or as the silver bullet and it’s really well known and it’s prescribed more often than ibuprofen*. (Caregiver/Site A/Non-crossover2)

There is also a common perception that paracetamol is safer than ibuprofen, even though evidence shows that safety of both medications is comparable [5].

*I’m under the understanding that paracetamol has a better safety profile than ibuprofen*. (HCP/Primary care/NP)

Therefore, it is widely available in most homes and healthcare settings.

*It’s just generally what I hear is always have Pamol, even the doctors have said it – what family doesn’t have Pamolin their house?* (Caregiver/Site B/Non-crossover1)

*So, there’s standing orders for paracetamol but we don’t have anything with ibuprofen*. (HCP/Primary care/NP)

### Trusting relationships

Having trusting relationships is key in enabling caregivers and HCPs to challenge historical ideas. Three focused codes comprised the trusting relationships category (Table 2; Additional file 3: Table S2).

#### Trusting the research process

When caregivers are approached by the PIPPA Tamariki research staff, trusting the research process allows them to see past their historical preconceptions and enrol their infant into the trial. Caregivers trusted the research staff and valued staff availability to be contacted for support during the trial.

*…just trusting in the process…So, with the study on ibuprofen I think the contact that we’ve had with the researchers or the people that have contacted me in terms of the study or um, yeah being available to ask any questions was, um, settling, it was good…or if she had any side effects, being able to contact people, you know, people would follow-up*. (Caregiver/Site A/Non-crossover2)

Among HCPs, trusting the requisite ethical and regulatory processes that govern the conduct of research was essential for their endorsement of the trial.

*I would have thought if you’re doing a study that has some children randomised to ibuprofen then that you would have gone through appropriate ethics approval and that it would be safe and appropriate for that age group to be randomised to that, so, I wouldn’t have any opposition to that at all*. (HCP/Primary care/NP)

Building strong trusting relationships with research staff was crucial for caregivers to be committed to the research, motivating them to adhere to ibuprofen.

*I told everybody because they were all trying to give him Pamol. It was like, he can’t have that so he’s actually in a study, like I had to remind them all the time. I told them he’s on the PIPPA study. I had to say every time I went to the doctors, over and over and over…Yeah, I had to. I didn’t want to like ruin the study.* (Caregiver/Site B/Crossover2)

#### Trusting healthcare professionals

Caregivers expressed their complete and implicit trust in HCPs with whom they have a long-standing relationship. Caregivers’ trusting relationships with their usual HCPs reinforces their trust in the research process.

*I had a chat with our paediatrician and he’s* [paediatrician’s name]*, he’s a rockstar – he didn’t have an issue with it and I sort of trust everything that he says*. (Caregiver/Site A/Crossover1)

#### Being accepting

When trusted HCPs are being accepting and supportive of caregivers’ decision to participate in the PIPPA Tamariki trial and to use ibuprofen in preference to paracetamol, caregivers found it reaffirming and supported their efforts to avoid trial medication crossovers.

*…there was a doctor and he actually gave, he actually reaffirmed what I was doing and what I was thinking as well. And he said, look there’s nothing you can do – you can give them Pamol* [in addition to ibuprofen] *but what they really need is a hug and yeah just to calm them down and just you and there I go and so it was good confirmation.* (Caregiver/Site A/Non-crossover2)

Caregivers’ families were also an important source of reaffirmation and support.

*Um they were actually they were interested in the trial. My family, we had no problems with it because my brother is an asthmatic and so for us it was, if anything to help another person who is either asthmatic or anything of those things in the trial was designed to support, they were all positive with. So yeah, it was very positive, responsive support we got*. (Caregiver/Site C/Non-crossover1)

When asked what made it easier to adhere to ibuprofen during the trial, caregivers discussed the value of being collaborative with their families through the process of shared decision-making.

*I think it has helped that my husband has been quite on board with doing the ibuprofen. So, I think, you know, you both need to be on the same page with that.* (Caregiver/Site B/Non-crossover1)

### Being discerning

Once trusting relationships have been forged, we can facilitate the process for caregivers and HCPs to be discerning of new information they receive when juxtaposed with their historical knowledge and beliefs. One caregiver aptly summarised the process of being discerning:

*You’re just winging it really, hoping for the best and trying to listen to everyone’s opinions but also make your own informed opinions*. (Caregiver/Site B/Non-crossover1)

Three focused codes comprised the being discerning category (Table 2; Additional file 3: Table S3).

#### Raising awareness

Raising awareness among participants regarding evidence-based and appropriate use of paracetamol and ibuprofen can occur in a number of ways. Caregivers and HCPs discussed how the PIPPA Tamariki trial prompted them to seek out information in order to raise their own awareness:

*I’ve never taken ibuprofen so I was more inclined to go with paracetamol or Pamol*. *But when I was chosen for ibuprofen, I had a read up about it and I wasn’t too concerned so that was fine.* (Caregiver/Site A/Crossover1)

Often, HCPs were also in a position to raise awareness about paracetamol, ibuprofen and fever management practices among their patients.

*I’m pretty hot on, you know, advising parents that actually giving paracetamol, ibuprofen to bring down a fever doesn’t actually stop the febrile convulsion*. (HCP/Primary care/NP)

#### Being confident

Equipped with knowledge and information, participants gain confidence to “*keep up with the times*” (Caregiver/Site B/Non-crossover2) and be less fearful of ibuprofen.

*I’m okay* [with using ibuprofen] *because times are changing…Yeah so, we’re not always going to go back to the like old school remedies or things like that*. (Caregiver/Site B/Non-crossover2)

When friends and family express opposing views, often based on historical preconceptions, several caregivers demonstrated being confident and resolute in their commitment to the research:

*I think it was more the older generation that were sort of like, oh that’s interesting, why are you doing that, type thing… one would be the mother-in-law. So, she’s done nursing and things like that as her previous roles. So, I thought that her opinion was quite interesting… she obviously really likes Pamol so (laughs)… I guess um for me, like I know and respect her opinion but also… I know that, as time goes on, there’s always research done and things change… she did sort of raise a little bit of a question mark. Um and I guess we took it on board but we were happy to be a part of this* [the PIPPA Tamariki trial]*.* (Caregiver/Site B/Non-crossover1)

#### Being discerning about COVID-19

The trusting relationships that have been established, together with the awareness and confidence that have developed, also allowed caregivers to be discerning when information about the potential harm of ibuprofen in COVID-19 was released in the media. We found that caregivers did not have “*a knee-jerk reaction*” (Caregiver/Site C/Non-crossover2) to COVID-19 based on media reports, because they trusted their the research process implicitly.

*If I’m honest, I thought that it was just rubbish. I didn’t think that this COVID-19 had come out and that suddenly this really reputable well-known medicine* [ibuprofen] *could have such a detrimental effect on anyone. We’d made the decision to keep using it and we did keep using it and yeah, he didn’t die so (laughs). Yeah that wasabonus*. (Caregiver/Site C/Non-crossover2)

### A condition – being conflicted

Having established trusting relationships that empower caregivers and HCPs to challenge historical ideas and to become more discerning, the question remains: why do crossovers occur? The following memo written by the first author illustrates her developing theoretical sensitivity to the key condition of being conflicted, leading to an emotional response underlying the crossover events (Additional file 3: Table S4):

*Being conflicted usually happens when they see a sick/miserable/hot baby, and often this can be additionally influenced by other parents or HCPs. When there is an internal conflict, emotion takes over (including historical fever phobia) and all reasoning goes out the window.* (Memo/1 March 2021/ET)

#### Seeing a sick baby

Caregivers of infants who had a crossover event often described a sense of desperation when faced with their ill-appearing baby and felt the need to “*do whatever* [they] *can do to help* [their baby] *feel better*” (Caregiver/Site A/Crossover1).

*She was a really, really unsettled baby, like a really unsettled baby… she was just crying all day and all night. And I think I was pretty desperate so that was the first time that she was given Pamol and I don’t think it made much of a difference to her crying… but we were so desperate so we thought, oh well, we’ll try anything*. (Caregiver/Site C/Crossover1)

HCPs experience a similar internal conflict when faced with an ill-appearing patient:

*If they were, you know, flat out on their back and didn’t move for three hours and weren’t drinking, I’d probably break down and give it* [paracetamol]. (HCP/Hospital-based/Senior ED doctor)

#### Feeling pressured

Being conflicted can be intensified when participants feel pressured to revert to historical, non-evidence based practices. Caregivers described feeling pressured by other family members or even by their trusted HCPs:

*I felt a bit crap because, you know, we’re in this trial and we believe in science and sort of really wanted to stick to it* [ibuprofen]. *But sort of weighing that up against the fact well, you know, on the other hand I’ve got another health professional telling me that I should try something* [give paracetamol] *and I was pretty desperate so*. (Caregiver/Site C/Crossover1)

Conversely, some HCPs discussed feeling pressured by both caregivers and colleagues to treat an ongoing fever:

*Like you should be encouraging yeah to go by the behaviour of the child as well. And with that, sometimes it depends on the parents a wee bit, how accepting they can be of that. Like some are so concerned about the fever, they don’t look at the child, like as a whole… Yeah there’s a wee bit from parents but there’s also sometimes from the doctors as well because you can have a child who’s febrile and tachy*[cardic] *and then, you know, I can remember going through a period where we really pushed not givingantipyretics if a child was happy and that. But then the doctors… they wanted to bring the fever down and see if there was still tachycardia. So, I felt I was losing that side of the argument so I probably don’t push it as much as I used to*. (HCP/Hospital-based/Senior ED nurse)

## Discussion

This study used constructivist grounded theory methodology to explore factors that influence the decision-making process when prescribing or administering ibuprofen in infants, and identified three categories: historical, trusting relationships, being discerning; and the basic social process of prioritising infant welfare, which accounted for crossover events when caregivers and HCPs are under the causal condition of being conflicted. Our study has generated an understanding of participants’ motivations for medication non-adherence in RCTs involving infants. As poor medication adherence in clinical trials remains an ongoing problem internationally with considerable implications [13], our findings are likely to be of relevance to many researchers.

It was not surprising to find that many participants held historical beliefs and engaged in non-evidence-based practices based on irrational fears regarding the consequences of fever, termed fever phobia. Fever phobia has been well-described over the last four decades [37–39], and a recent systematic review found that fever phobia continues to be a worldwide phenomenon affecting both caregivers and HCPs [40]. Many caregivers report using alternating or combination antipyretic regimens and worryingly, as our study found, this practice is often based on advice from treating HCPs [21, 41, 42], despite a lack of evidence to support this practice and the possibility of placing children at undue risk of toxicity from drug errors [21, 41–44]. While it is tempting to call for effective educational strategies, our study suggests that addressing the lack of knowledge in isolation may be insufficient to influence behaviour.

Our study highlights the crucial role that trusting relationships between participants and researchers played in challenging historical preconceptions, and as a prerequisite to raising participant awareness, gaining confidence and promoting adherence in the context of a paediatric RCT. Literature on clinical trial adherence in the paediatric age group is scarce. A qualitative study by Luchtenberg *et al* [45]*,* which explored children’s perceptions of the child-doctor relationship in research participation, found that familiarity and a trusting relationship led to a feeling of mutuality and enhanced children’s confidence, improved recruitment, and engagement throughout the research process. When their participants did not have a prior relationship with a researcher, as is the case with the PIPPA Tamariki trial, participants depended more on family support when introduced to research [45]. Several authors have also emphasised the importance of establishing participant trust [20], raising awareness [12, 13, 20], and involving family members or other social support networks [12, 20] to promote involvement in all aspects of the research process, including intervention adherence [12, 13], as well as participant recruitment [17–19] and retention [19, 20].

Caregivers valued the support they received from their treating HCPs with whom they have a trusting relationship, in their decision to enrol into the trial and adhere to their assigned intervention. Children interviewed by Luchtenberg *et al* [45] said they trusted their doctor who knew about their specific situation and were in a position to provide support and care. This supports findings from Dekking *et al* [46] in which parents said they valued the involvement of their child’s physician in the informed consent process. Given the level of trust research participants place in their treating HCPs, establishing relationships between researchers and treating HCPs to garner their support has been suggested as a strategy to enhance adherence [12]. Building strong relationships and coordination with primary care physicians and nurses in trusted community settings were found to be effective strategies in the recruitment and retention of socially disadvantaged participant groups often considered “hard-to-reach” [19, 47]. Our study suggests that such a strategy may also be useful to promote adherence in paediatric RCTs. The role of trusting relationships between researchers and treating HCPs warrants further investigation.

An unexpected finding was that our participants were not unduly influenced by social and news media warnings regarding ibuprofen use in the setting of COVID-19. Their response can be explained by the basic social process of prioritising infant welfare in action: strong trusting relationships with researchers enabled many caregivers to be discerning in the face of large amounts of information about COVID-19, which in turn enabled them to feel confident and safe to continue using ibuprofen. The same basic social process can inform strategies for reducing medication crossovers in the PIPPA Tamariki trial. In order to effectively educate caregivers and dispel historical myths and ideas, developing trusting relationships between researchers and caregivers is crucial. Strategies to build such trusting relationships may include frequent personalised contact with caregivers and their social support network, and having access to researchers particularly at times when they may feel conflicted about using ibuprofen. Ensuring caregivers’ trusted sources of information have up-to-date, evidence-based knowledge is essential so that they can support researchers to increase caregivers’ confidence. We need to help both caregivers and treating HCPs redefine their new place of safety that aligns with best practice guidelines (such as giving paracetamol/ibuprofen for infant distress rather than solely for temperature reduction, and avoiding alternating/combined antipyretics), so that they equate following best practice guidelines to prioritising infant welfare and do not feel conflicted in doing so.

Basic social processes are not present in all grounded theories, but when one is apparent they give the feeling of movement over time and can have general implications outside the confines of a single study [48]. Our results may also have implications in the clinical setting, or in situations where research and clinical care are combined. In both clinical practice and research, adherence to regimens is a challenge. The literature supports comprehensive interventions addressing cognitive, behavioural and affective aspects rather than single-focus approaches to promote clinical adherence [12, 49]. That our grounded theory addresses each of these components suggests that our findings may also relate to the broader topic of adherence.

Strengths of our study include the use of investigator triangulation, data source triangulation, and memo writing to develop a comprehensive narrative and enhance trustworthiness of the data. Identification of a basic social process increases the transferability of our findings to other contexts such as recruitment and retention in clinical trials, as well as adherence in the clinical therapeutic setting, adding value for researchers and clinicians alike.

Inevitably, our study had several limitations. Findings report only the experiences of caregivers and HCPs who were interviewed. Although planned, we did not undertake theoretical sampling of participants. However, we theoretically sampled by progressively and systematically tailoring interview questions for the purpose of explicating and refining the constructed theory, and we are reassured that theoretical saturation was reached. HCPs who prescribed medication crossovers are a potentially rich source of data, but it was pragmatically difficult to identify them from the trial database. Notwithstanding, negative cases such as HCPs and caregivers who were not involved in medication crossovers are vital to the process and their inclusion can lead to more sophisticated and nuanced theories [29]. As with any grounded theory study, different researchers may have constructed alternate codes and categories from the data.

## Conclusion

Using constructivist grounded theory, factors influencing ibuprofen use in infants and crossover events were identified. These factors contribute to the basic social process of prioritising infant welfare, which can inform strategies to promote adherence in the PIPPA Tamariki trial. Future studies should explore intervention adherence in the context of paediatric RCTs, and the role of trusting relationships between researchers and treating clinicians when conducting research.

## Supplementary Information

**Additional file 1.** Standards for Reporting Qualitative Research checklist v2 2022.02.10.

**Additional file 2.** Interview guide.

**Additional file 3.** Representative quotations.

## Data Availability

The datasets generated and/or analysed during the current study are not publicly available due to persons being potentially identifiable. Summary data are available from the corresponding author on reasonable request.

## Abbreviations

COVID-19: Coronavirus disease 2019
ED: Emergency department
GP: General practitioner
HCP: Healthcare professional
NP: Nurse Practitioner
PIPPA Tamariki: Paracetamol and Ibuprofen in Primary Prevention of Asthma in Tamariki
RCT: Randomised controlled trial
SRQR: The Standards for Reporting Qualitative Research
WHO: World Health Organization
