# Supplementary material for: Panic or peace – prioritising infant welfare when medicating feverish infants: a grounded theory study of adherence in a paediatric clinical trial
Source: BMC Pediatr. 2022 Apr 11;22:195. doi: 10.1186/s12887-022-03230-4 (PMC8995914; doi:10.1186/s12887-022-03230-4)
Supplement: Supplementary file 2 — Additional file 2. Interview guide. [file 12887_2022_3230_MOESM2_ESM.docx]

**Additional file 2. Interview guide**

| **Topic** | **Caregivers** | **Healthcare professionals** |
| --- | --- | --- |
| Introduction | Thank you for agreeing to be interviewed for this study.  Can you confirm that you are still happy to proceed with the interview?  You do not have to answer every question and can stop the interview at any time.  Before I start, do you have any questions you would like to ask me? | Thank you for agreeing to be interviewed for this study.  Can you confirm that you are still happy to proceed with the interview?  You do not have to answer every question and can stop the interview at any time.  Before I start, do you have any questions you would like to ask me? |
| Beliefs and attitudes about fever or pain medications | Tell me what your plans were for giving your baby medications for fever or pain before you were approached about the PIPPA Tamariki study?   - Specifically cover paracetamol, ibuprofen, what happened for other children (if any) - Why? Who or what influenced you?   What was your perception of ibuprofen when you enrolled your baby into the PIPPA Tamariki study?   - Why or how did you come to this view? | Tell me about your usual practice for prescribing/using analgesia or antipyretics to infants <1 year.   - Why/what is the basis for this practice? - Where/how do you get guidance for this practice?   What is your perception of **ibuprofen** use:   - In infants - In infants <6 months - In infants <3 months - In mothers who are breastfeeding? - Why? What is the basis of this perception?   What is your perception of **paracetamol** use in babies?   - In infants - In infants <6 months - In infants <3 months - In mothers who are breastfeeding? - Why? What is the basis of this perception?   Tell me about the advice you give parents/guardians of infants <1y about the use of analgesia or antipyretics |
| Being on the PIPPA ibuprofen treatment arm | How did you feel when your baby was randomised to ibuprofen?  What did health professionals say when they heard your baby was taking ibuprofen?  What did your family and friends say when they heard your baby was taking ibuprofen?  Tell me about your experience with giving your baby ibuprofen during PIPPA T study?  If crossover:  Tell me about the first time your baby was given **paracetamol** instead of ibuprofen   - Circumstances - Reasons - Others involved in decision-making? - How did you feel about the decision to use the ‘other’ medicine? - Given as alternating regimen, combination, or instead of? | How did you/would you feel about a patient <1y in your care being randomised to ibuprofen in a trial? |
| COVID-19 | Were you aware of the concerns initially raised about using ibuprofen to treat symptoms of COVID-19 (from WHO, other international bodies, the lay press releases)?   - If yes, how did you hear about these concerns? (e.g. social media, news, friends, health professionals? - Did you try to find out more / do your own research about these concerns? How? What sources? - What sort of effect did these concerns have on your comfort about using/prescribing ibuprofen to your patients? - Now that this appears to be resolved, how do you feel about giving ibuprofen to your baby now? | Were you aware of the concerns initially raised about using ibuprofen to treat symptoms of COVID-19 (from WHO, other international bodies, the lay press releases)?   - If yes, how did you hear about these concerns? (e.g. social media, news, friends, health professionals? - Did you try to find out more / do your own research about these concerns? How? What sources? - What sort of effect did these concerns have on your comfort about using/prescribing ibuprofen to your patients? - Now that this appears to be resolved, how do you feel about using ibuprofen for your patients now? |
| Theoretical sampling – additional lines of inquiry | Explore fever phobia vs pain/distress/misery  Febrile convulsions  Information sources | Explore fever phobia vs pain/distress/misery  Febrile convulsions  Information sources  Safe practice / following guidelines  Availability of ibuprofen on standing orders or in cupboards |
| Demographics | Already collected as part of PIPPA Tamariki enrolment | Age, qualifications, years of practice |
