# Supplementary material for: Panic or peace – prioritising infant welfare when medicating feverish infants: a grounded theory study of adherence in a paediatric clinical trial
Source: BMC Pediatr. 2022 Apr 11;22:195. doi: 10.1186/s12887-022-03230-4 (PMC8995914; doi:10.1186/s12887-022-03230-4)
Supplement: Supplementary file 3 — Additional file 3. Representative quotations. [file 12887_2022_3230_MOESM3_ESM.docx]

**Additional file 3. Representative quotations**

**Table S1. Initial codes, focused codes and representative quotations for *historical* category**

| **Focused codes** | **Initial codes** | **Caregiver** | **HCP** | **Representative quotations** |
| --- | --- | --- | --- | --- |
| Historical | Historical | X  X  X | X  X  X  X  X  X | *Well I think historically, when the girls were younger, you couldn’t give Brufen below the age of six months.* (Caregiver/Site A/Crossover2)  *When I was a baby – Pamol was the main form of medication and it was so, I think it’s probably just information that’s been passed down and so that’s how I came to the consensus.* (Caregiver/Site C/Non-crossover1)  *We’ve just grown up in such a paracetamol driven environment but maybe ibuprofen was the after-thought or the additional think you would take if you weren’t feeling well.* (Caregiver/Site C/Non-crossover2)  *It comes from experience and learning, the papers that we’ve done um just I guess it’s just what’s out there… just because it’s what we’ve always done.* (HCP/Primary care/NP)  *Just because we grew up with Pamol, pretty much. I would have followed what my Mum did with us and my nieces and nephews and used Pamol. That’s been a family thing.* (Caregiver/Site B/Non-crossover2)  *I suppose because when I started practising back in the mid-90s, ibuprofen was not given*. (HCP/Hospital-based/Senior ED doctor)  *When I first and I practiced, you know, it was unsafe, well not unsafe, it wasn’t deemed practice to administer ibuprofen to young babies.* (HCP/Hospital-based/Senior ED nurse)  *I think for years, we’d been told you can’t give it to children.* (HCP/Hospital-based/Senior ED nurse)  *So, my bias is probably to prescribe more paracetamol because that’s what I’ve done for years.* (HCP/Primary care/Senior GP) |
| Lack of knowledge | Lack of knowledge | X  X  X  X | X  X | *I just assumed that Nurofen [ibuprofen] was a medication only suitable for children of a higher age. Like I didn’t know what it was something that was able to given to babies.* (Caregiver/Site C/Non-crossover2)  *I’d heard about it [ibuprofen], just not for like really small babies. I’d heard about it for like younger children and up, not for really small babies.* (Caregiver/Site B/Crossover1)  *I didn’t know that kids can take Ibuprofen.* (Caregiver/Site B/Crossover2)  *I actually wasn’t aware that you could give it to younger, younger kids or even to newborns. Yeah, so that was my perception was ibuprofen is for older and Pamol and that is suitable for younger.* (Caregiver/Site C/Non-crossover1)  *I don’t know what age ibuprofen can be given down to*. (HCP/Primary care/Practice nurse)  *I think I always thought that Ibuprofen doesn’t help with the temperature – it’s more like pain.* (HCP/Hospital-based/Paediatric ward nurse) |
|  | Paracetamol works better | X |  | *Paracetamol was better for high fevers and ibuprofen was more, I always thought a better pain killer.* (Caregiver/Site C/Crossover2) |
|  | Ibuprofen works better |  | X  X | *Some families may not use paracetamol because they don’t believe it works.* (HCP/Primary care/Practice nurse)  *Sometimes people won’t even bother with Pamol because they know ibuprofen works better for their child so they’ll just as for that.* (HCP/Hospital-based/Paediatric ward nurse) |
|  | Immunisation |  | X | *The nurses can actually give out a little bottle of paracetamol with vaccinations if the doctor hasn’t done it as part of that consultation.* (HCP/Primary care/NP) |
| Fever phobia | Fever phobia | X | X  X | *Like I said, they would cook, like they’d be up near 40 [degrees Celsius] and I was petrified, probably overly so as a neurotic nurse. And I would do anything I could to bring it down.* (Caregiver/Site A/Crossover2)  *Um so probably over the 38, 38.5 [degrees Celsius], yeah would be worrying, yeah.* (HCP/Hospital-based/Paediatric ward nurse)  *If they are quite hot, I think it’s a good idea to bring their fever down.* (HCP/Primary care/Senior GP) |
|  | Alternating antipyretics | X | X  X | *…they were really unwell and that’s when we’ve taken them into hospital and they said right, you know, it’s a fever – you can give them paracetamol and then two or three hours later give them the ibuprofen and so that way if you give them both together, they both wear off together. You can stagger the doses and that’s when I first learnt that you could stagger them and have them together.* (Caregiver/Site C/Crossover2)  *I’m really comfortable about them alternating for fever in order to cover the 24 hours… give a dose of paracetamol and if the fever’s not settled in 30 minutes or so, add in a dose of ibuprofen.* (HCP/Primary care/Senior GP)  *I would usually tell them to use one or the other and their temperature hasn’t come down within a couple of hours, then give them the other one.* (HCP/Hospital-based/Senior ED doctor) |
|  | Combined antipyretics | X | X | *You feel like you’re doing something more when you give both of them.* (Caregiver/Site C/Crossover1)  *Giving paracetamol and ibuprofen together one time and that just helps to bring it down to within, look I don’t know what you call, a manageable level.* (HCP/Hospital-based/Senior ED nurse) |
|  | Convulsions | X |  | *Ah convulsions was my biggest worry.* (Caregiver/Site A/Crossover2) |
| “Paracetamol is the go-to” | Paracetamol is the go-to or first-line |  | X  X  X | *Paracetamol would have just been the one that you would have gone to.* (HCP/Hospital-based/Senior ED doctor)  *I would tend to give paracetamol first and then if they still seemed to need more pain relief, I would give them like an ibuprofen as a secondary.* (HCP/Hospital-based/Senior ED doctor)  *I don’t mind ibuprofen, I don’t have a problem with it and I think I just go for paracetamol first but I don’t know why really (laughs). It’s just kind of like a subconscious thing maybe that I do.* (HCP/Hospital-based/Paediatric ward nurse) |
|  | Branding | X |  | *And you know, because Pamol’s been prescribed for so many years, I think people just gravitate to Pamol as the medication of choice or as the silver bullet and it’s really well known and it’s prescribed more often than ibuprofen.* (Caregiver/Site A/Non-crossover2) |
|  | Paracetamol is safe |  | X  X  X | *My understanding is that [paracetamol is] possibly safer for infants to have than ibuprofen but I don’t actually know that guaranteed.* (HCP/Primary care/Practice nurse)  *I’d be comfortable with either [paracetamol or ibuprofen], yeah. I think we still have; I think we still have a feeling in myself that paracetamol’s safer.* (HCP/Primary care/Senior GP)  *Maybe it’s not actually correct or not but, you know, do know what the side-effect profile is of ibuprofen and um that it can be quite significant and paracetamol um obviously there is a side-effect profile as well but if it’s taken correctly it’s less likely to cause problems than ibuprofen in normal doses can still cause the side-effects,* (HCP/Primary care/NP) |
|  | Availability and accessibility | X  X |  | *Pamol was the top thing to give your kids and you always had it in the cupboard.* (Caregiver/Site B/Non-crossover1)  *I’ve always just bought Pamol from the shop if I needed it. I always have some in the cupboard just in case.* (Caregiver/Site A/Crossover1) |

**Table S2. Initial codes, focused codes and representative quotations for *trusting relationships* category**

| **Focused codes** | **Initial codes** | **Caregiver** | **HCP** | **Representative quotations** |
| --- | --- | --- | --- | --- |
| Trusting the research process | Trusting the research process | X  X |  | *I thought I would just trust yous knowing that you’re going to give it to a baby and yeah you had my trust in that.* (Caregiver/Site B/Non-crossover2)  *With the trial, I mean, we’re dealing with health professionals so there is a going to be a level of trust, right?* (Caregiver/Site A/Non-crossover2) |
|  | Being committed | X |  | *We’re in this trial and we believe in science and sort of really wanted to stick to it.* (Caregiver/Site C/Crossover1) |
| Trusting healthcare professionals | Trusting healthcare professionals | X  X  X |  | *I think whether it’s a doctor or a nurse or the study, it’s at the end of the day, for me, you guys are health professionals, you know, and you’re good at what you do. So, it’s not that I trust one over the other but as a whole, that’s where the trust is.* (Caregiver/Site A/Non-crossover2)  *I think there’s just that trust factor, you know, I think with (older child) I trusted, there was a lot of trust in what the doctors are saying and what the doctor prescribed because I don’t know what I don’t know.* (Caregiver/Site A/Non-crossover2)  *We always continue to talk to our GP throughout any time I was feeling like maybe we need to give her any medication, I’d always consult with my GP to make sure it was appropriate to give it to her.* (Caregiver/Site C/Non-crossover1) |
| Being accepting | Being accepting and supportive | X  X  X  X |  | *Our GP or one of the GPs had heard of the trial and so was like, oh that’s cool – it’ll be interesting to see what the results are.* (Caregiver/Site C/Non-crossover1)  *We went to the GP the other day and she’d obviously had some kind of alert about it on her computer system so she knew about it.* (Caregiver/Site C/Crossover1)  *When we were in hospital with him and we said, he’s part of the PIPPA study and we were in there a couple of days so he’s on ibuprofen and they just said, yeah cool and didn’t bat an* *eyelid.* (Caregiver/Site C/Crossover2)  *My mum is really the only person who had a strong opinion that she was obviously the one that was with me when I was in and out of hospital as a baby so she was all for it. She was like I can’t believe that it’s taken this long to there even to be a discussion about it.* (Caregiver/Site C/Non-crossover2) |
|  | Being collaborative | X |  | *If something comes up, we talk about it, how we feel and then that information helps us decide things together.* (Caregiver/Site A/Non-crossover1) |

**Table S3. Initial codes, focused codes and representative quotations for *being discerning* category**

| **Focused codes** | **Initial codes** | **Caregiver** | **HCP** | **Representative quotations** |
| --- | --- | --- | --- | --- |
| Being discerning | Being discerning | X  X |  | *Take everything they say with a grain of salt – put it all in the cloud and take out what I want.* (Caregiver/Site A/Crossover1)  *Like don’t trust word of mouth and stuff – brain of your own, think for yourself, go to the proper places.* (Caregiver/Site A/Non-crossover1) |
| Raising awareness | Raising parent awareness | X |  | *We had no issues with it [ibuprofen] whatsoever because we’d been talked through that it was similar in terms of what it can offer in terms of medication so we had no issues of her being on either of the trial medications.* (Caregiver/Site C/Non-crossover1) |
|  | Raising HCP awareness |  | X  X  X | *I was probably influenced by you guys doing the PIPPA study… made me feel much safer about doing it [prescribing ibuprofen to infants]. And so, you know, I did a little check myself… there’s a Health Navigators site about ibuprofen for little ones…and so that made me feel a lot more comfortable… You sent a decent letter out when you sent information out…* (HCP/Primary care/Senior GP)  *Like I probably thought like a fever, being miserable kind of go hand in hand, you know. Whereas now, I know you can have a fever but you can be happy, you know, like children yeah.* (HCP/Hospital-based/Paediatric ward nurse)  *Having the PIPPA study was wonderful, you know, to bring research out and to move different things forward.* (HCP/Hospital-based/Senior ED nurse) |
| Being confident | Being confident | X |  | *I’m the sort of person that actually if they had, had thoughts, I probably would have told them to just keep them to themselves anyway.* (Caregiver/Site A/Crossover1) |
|  | Keeping up with the times | X  X |  | *I’d like to keep up with the times and move with it and if these are the new options that we have, then I’ll try it*. (Caregiver/Site B/Non-crossover2)  *So, I know that, as time goes on, there’s always research done and things change.* (Caregiver/Site B/Non-crossover1) |
| Being discerning about COVID-19 | COVID-19 | X  X | X | *…had a look at what those were and whether we still felt comfortable proceeding. And from the readings, me and my partner were still quite happy to continue with the trial so we* *did.* (Caregiver/Site C/Non-crossover1)  *I guess it just frustrated me that there could be such a flipflop in attitude. Well, not attitude but Covid-19 is still so new. I mean, we’re only one year really and we’re into this pandemic. That’s probably going to last a few more years and I just felt that maybe the reaction was a little bit knee-jerk. And I just thought, you know, I’m really relieved we didn’t make a knee-jerk reaction as well and just kind of stuck with what our gut told us and that was that this reputable drug or whatever, was probably fine and this one random doctor causing a bit of a stir somewhere. I mean, because if people told you not to eat bananas during the pandemic, you wouldn’t [not] eat bananas, right. You know, like it’s just a bit of scaremongering in a way.* (Caregiver/Site C/Non-crossover2)  *Here were a couple of very small studies that came out of somewhere that showed that maybe there was some issue with ibuprofen. I figured, like all new diseases, there was so much unknown about it, that a couple of cases aren’t going to necessarily change the world… I mean, to me, everything that came out about the pandemic, I would only really be interested in the research starting about now because it was just too early. You know, so there was a month’s worth of information – how can you make a reasoned decision based on a month’s worth.* (HCP/Hospital-based/Senior ED doctor) |

**Table S4. Initial codes, focused codes and representative quotations for *being conflicted* condition**

| **Focused codes** | **Initial codes** | **Caregiver** | **HCP** | **Representative quotations** |
| --- | --- | --- | --- | --- |
| Seeing a sick baby | Seeing a sick baby | X  X  X |  | *I had no choice because he was really ill.* (Caregiver/Site B/Crossover2)  *I hope this doesn’t stuff the study too much but he needs it.* (Caregiver/Site C/Crossover2)  *We were so desperate so we thought, oh well, we’ll try anything.* (Caregiver/Site C/Crossover1) |
| Feeling pressured | Pressure from HCPs | X |  | *I guess also that hard part is the doctor as well and when a doctor’s telling you, someone might need, first time mum, like why don’t you have Pamol or actually take both of them, then that’s what you do. You do what your health professional tells you to do.* (Caregiver/Site B/Non-crossover1) |
|  | Pressure from parents | X |  | *I still have to give it because my mum said if I don’t, like I don’t know if his head’s pounding or he feels sore, so she just told me to give it.* (Caregiver/Site B/Crossover2) |

COVID-19: Coronavirus disease 2019

ED: Emergency department

GP: General practitioner

HCP: Healthcare professional

NP: Nurse practitioner

PIPPA: Paracetamol and Ibuprofen in Primary Prevention of Asthma
